# Sustainable Health Development Goals (SHDG): breaking down the walls

**DOI:** 10.11604/pamj.2015.22.306.6468

**Published:** 2015-11-26

**Authors:** Obinna Ositadimma Oleribe, Mary Margaret Elizabeth Crossey, Simon David Taylor-Robinson

**Affiliations:** 1 Excellence & Friends Management Care Centre (EFMC) Abuja Nigeria; 2 Hepatology Unit, Imperial College London, 10th Floor, QEQM Building, St Mary's Hospital Campus, South Wharf Road, W2 1NY, London, United Kingdom; 3 Department of Medicine, Jos University Teaching Hospital, 2 Murtala Mohammed Way, Jos, Plateau State, Nigeria

**Keywords:** Sustainable Development Goals, Millennium Development Goals, vertical, integrated

## Abstract

The world's governments failed to achieve the *Health for All 2000* goals from the Alma Ata Declaration of 1978. Although a lot of milestones have been covered since 2000, the world's governing authorities are unlikely to achieve the current Millennium Development Goals (MDGs) which expire by the end of this year. The inability to achieve these goals may be linked to the multiplicity of health-related directives and fragmentation of health systems in many countries. However, with the proposed 17 sustainability development goals, health has only one universal aim: to ensure healthy lives and promote wellbeing for all at all ages. Accomplishing this will require a focus on health systems (system-thinking), commonization of services and full integration of services with total dismantling of vertical programs across the world.

## Commentary

In 1978, the governments around the world came together to develop and accept the primary health care concept as the basis for health for all by 2000 [1]. However, the balkanization of the process led to selective primary health care services aimed at achieving immediate short-term gains and this diverted focus and loss of momentum with resultant inability of the world to achieve the set goal of *“Health for All”* by 2000 [2–4]. When it became a reality that “Health for All” would not be achieved by 2000, world leaders took another proactive step to develop the Millennium Development Goals (MDGs). These were designed to eradicate some common diseases, minimize health inequality, improve environmental health and build global partnerships [5]. MDGs have led to global improvement in health outcomes, with some nations meeting and exceeding the set targets [6]. However, as 2015 - the end year of MDGs - draws to a close in the next few months, it is becoming apparent that many countries of the world, especially those in sub-Saharan Africa may never achieve these goals [5–8]. These deficiencies spurred world leaders to develop the proposed Sustainable Development Goals (SDGs), which are expected to replace the MDGs by the end of the 2015 [9]. The SDGs have come a long way and for the next 15 years (2015 - 2030), will be the primary focus of global authorities. Recently, the proposed 17 goals were unveiled. These are: (1) To end poverty in all its forms everywhere; (2) To end hunger, achieve food security and improve nutrition and promote sustainable agriculture; (3) To ensure healthy lives and promote wellbeing for all at all ages; (4) To ensure inclusive and equitable quality education and promote lifelong learning opportunities for all; (5) To achieve gender equality and empower all women and girls; (6) To ensure availability and sustainable management of water and sanitation for all; (7) To ensure access to affordable, reliable, sustainable and modern energy for all; (8) To promote sustained, inclusive and sustainable economic growth, full and productive employment and decent work for all; (9) To build resilient infrastructure, promote inclusive and sustainable industrialization and foster innovation; (10) To reduce inequality within and among countries; (11) To make cities and human settlements inclusive, safe, resilient and sustainable; (12) To ensure sustainable consumption and production patterns; (13) To take urgent action to combat climate change and its impacts; (14) To conserve and sustainably use the oceans, seas and marine resources for sustainable development; (15) To protect, restore and promote sustainable use of terrestrial ecosystems, sustainably manage forests, combat desertification, and halt and reverse land degradation and halt biodiversity loss; (16) To promote peaceful and inclusive societies for sustainable development, provide access to justice for all and build effective, accountable and inclusive institutions at all levels; and (17) To strengthen the means of implementation and revitalize the global partnership for sustainable development (10).The goals address six primary themes of dignity, people, planet, partnership, justice, and prosperity for majority, reflecting the focus of the MDGs but with further disaggregation [10].

### The SDGs and Health Industry

The MDGs provided a focal point for governments and served as a foundation for policy development, and funding to end poverty by improving the lives of poor people [11]. They also provided a rallying point for NGOs to hold both government and funding organization accountable for public health outcomes. However, the eight MDGs failed to consider the root causes of poverty, gender inequality, and the holistic nature of development [11]. The report also emphasized the fact that the MDGs made no mention of human rights, did not specifically address economic development, and targeted very poor countries, using funds from wealthy countries. Unlike the MDGs that were criticized for being too narrow, the strength of the SDGs is that it is all inclusive having 17 goals and 169 targets. The SDGs cover key development issues, including root causes of poverty, gender, and sociocultural inequalities. Although all the goals will indirectly affect the health and well-being of people directly or indirectly, only one focuses on health - GOAL 3: **to ensure healthy lives and promote well-being for all at all ages**. This is unlike MDGs where three of the eight goals addressed health (i.e. MDG 3 - To reduce child mortality; MDG 4 -To improve maternal health; and MDG 5 -To combat HIV/AIDS, malaria, and other diseases). In the SDGs, there is just one goal focusing on health out of 17 goals. The question has to be asked as to whether this is a minus for health?

### Lessons from the past multiple goals resulting in vertical programs

The majority of previous attempts at making health and healthcare services available to the people, as and when they need them, failed. Why did these efforts at improving health fail to achieve the set targets, especially in sub-Saharan Africa? Were they over ambitious or could many goals resulting in verticalization of services have contributed to poor outcomes and ineffective reach/poor sustainability in the health improvement? Vertical programs have their advantages. However, they also hinder sustainable program development, especially in health. Atun, Bennett and Duran in 2008 revealed that vertical programs have limited benefits when compared with integrated delivery of health services [12]. Vertical programmes may be useful as a temporary measure when the health system is weak, and rapid response is needed which allows economies of scale in addressing the needs of particular target groups that may be difficult to reach [12]. Although vertical programs may yield immediate tangible benefits, such benefits are short-lived and unsustainable. Fragmenting health into vertical programs has not succeeded in building sustainable health systems globally. The need to proffer immediate solutions to specific disease conditions in specific populations, especially in middle and low-income countries, among women and children, and even in at-risk population have beyond delivery short-term benefits, over the years, weakened the healthcare systems. These vertical health care programs that have addressed health issues one disease at a time and to specific populations does not deliver long term benefits to population health, as they are grant policy limited and end the moment the funding stops. Current vertical programs usually resulting from external funds include programs targeting poliomyelitis, malaria, Guinea worm, tuberculosis, HIV/AIDS, vaccine-preventable diseases and vitamin A deficiency, amongst several others. While a number of these vertical programs have achieved a measurable level of success (like the smallpox war in late 1960 - 1970s), others have failed to make the expected impacts (such as the “Roll Back Malaria” program). Based on the perceived success of some disease-specific vertical programs globally, a new order of vertical programs emerged in the mid-twentieth century targeting specific populations/communities - women, children, and key populations, most at risk, etc. While these ideas were laudable in that they brought about immediate and identifiable reduction in morbidity and mortality within these populations in communities where these programs were either piloted or fully implemented, they all failed to produce sustainable change in the health and wellbeing of the communities.

### Moving forward: breaking down the walls

Previous healthcare goals, including the primary health care goals of 1978 and the MDGs of 2000, all*verticalized* health services, building silos within and across programs. These vertical programs focused on single diseases, specific groups of people or sometimes definite communities. They also developed disease or group-specific administrative structures, reporting channels, budget, accounting systems and personnel with very little integration into the larger health system [13, 14]. Most of them were short-term interventions and did not sustainably address poor populations overall disease burden. Although one disease might be controlled or eliminated, recipients of these interventions may often die from other diseases or their complications [14]. Despite the gains of vertical programs, for sustainable and health-wide interventions, there is the need to de-emphasize them actively and focus on building health systems that make sense. This is what SDGs seek to achieve. The single health goal of SDG is a call to a unified integrated health service delivery approach. This paradigm shift from multiple vertical programming goals should be seen as a golden opportunity to treat health and health related issues as a single holistic issue. It is time for all health care practitioners, policymakers, and public health advocates to fix the health system by working synergistically not tangentially towards better health outcomes globally. To realize the World Health Organization's (WHO) broad definition of health as “a state of complete physical, mental, and social well-being and not merely the absence of disease or infirmity”, every effort should be focused on the entire population, on all health compromising issues, and in all communities [15]. With just one health goal, all interested players will work together towards achieving this single goal by adding value to health in one way or the other. Governments of nations, particularly African countries, “donor” organizations such as PEPFAR, Global Fund, Clinton Foundation, UNIT One, etc.; foundations and interested individuals should unite for once to achieve a single goal that will change the entire global health outlook. Challenges of inefficiencies in resource management, duplication of efforts, establishment of parallel delivery structures, unskilled service delivery, fragmentation of the health system, missed opportunities to treat multiple issues in an integrated fashion [12, 13] will be reduced.

### Maximizing the SDGs opportunities for Health

SDGs call on policy makers and health workers to de-verticalize the health system destroys structural walls and dismantle all stand-alone programs. The single SDGs health goal requires that health practitioners the world over join hands to build a unified holistic health system. Vertical programs have given immediate gains that are unsustainable. Multiple goals have dispersed our energies leading to minimal long-term outcome. However, together, greater impact can be made, building healthier sustainable systems, reducing health inequality, improving equity in health distribution and positively affecting more lives. Everyone has a stake in the proposed SDGs, and everyone has a defined role to play. The government, private sector, educational institutions, establishments, non-governmental organizations, philanthropist, missionaries, health missions, farmers, civil servants, industrialist, students, unskilled workers have specific roles to play. Laudable as these goals are, if we fail to unite and play our parts, they will never be achieved. We should position ourselves to be part of this. With the signing of the SDGs document by the world, it has become the compass for global development for the next 15 years. Will the entire world fail again? Each of us can focus on one or more aspects of this health goal, innovate to make a difference in it; and stay committed until a difference is made. Using a system thinking approach, this is the time to build health systems in nations of the world with full commonization and integration of services. An effective interdisciplinary collaboration will be required.
